# Exercise-Induced Bronchoconstriction Among Adolescent Athletes With Asthma: A Systematic Review

**DOI:** 10.7759/cureus.40643

**Published:** 2023-06-19

**Authors:** Sawsan H Hashim, Miad I Alenezi, Rawan M Alenezi, Wafa T Alanazi, Mooj M Alruwaili, Almaha A Alali, Areej M Alanazi

**Affiliations:** 1 Department of Pediatrics, Northern Border University, Arar, SAU

**Keywords:** a systematic review, athletes, adolescents, asthma, exercise-induced bronchoconstriction

## Abstract

Exercise-induced bronchoconstriction (EIB) is a concern that frequently affects athletes and regular exercisers. The main objective of this systematic review is to study recently published literature that evaluated the risk of EIB among adolescent athletes with asthma. PubMed, Web of Science, Science Direct, EBSCO, SCOPUS, Wiley, and Cochrane Library were searched. Study articles were screened by title and abstract using Rayyan QCRI then a full-text assessment was implemented. A total of ten studies with 3129 adolescent athletic subjects were included in this review. The prevalence of EIB ranged from 2.1% to 61%. Most studies have demonstrated that athletes in their adolescence suffer from EIB, which requires regular management. Two studies have reported that low-income communities and humidity levels are risk factors for EIB. We found that EIB is frequent among adolescent athletes. The prevalence varies between countries due to different social and environmental factors.

## Introduction and background

Asthma is the greatest prevalent non-contagious disease in children and adolescents globally, affecting between 8% and 9% of children and teenagers under the age of 18 in developed nations and a growing number of children in low- and middle-income countries, according to estimates [1, 2]. The risk and severity of asthma in children and adolescents can be influenced by a wide variety of environmental, host/lifestyle, and genetic variables, which is recognized as a multifaceted disease [3]. One of the most frequent causes of bronchospasm in asthmatics is exercise and physical activity [4]. A fundamental characteristic of bronchial asthma, bronchial hyperreactivity, affects athletes more frequently than non-athletes, notably swimmers and participants in winter sports [5]. Exercise-induced bronchoconstriction (EIB) is one of the symptoms of activity-induced respiratory symptoms, which often entail abrupt constriction of the airways that happens during or after exercise [6]. But activity is also a well-known asthma trigger, and 35% of asthmatic kids report experiencing symptoms when exercising [7]. On the other hand, it has been demonstrated that patients with asthma can benefit from exercising. Exercise has been linked to improved cardiorespiratory fitness, according to recent evidence from controlled trials that used a physical training program intervention in children and adolescents with asthma (aged 6-18 years) [8]. In some instances, exercise can also lessen EIB. Furthermore, children who routinely exercise have been shown to have decreased prevalence of asthma symptoms when they have high levels of fitness [9]. EIB is characterized as the abrupt start of exercise-induced bronchoconstriction (during or soon following exercise) [10]. Although it has been estimated that up to 90% of individuals with underlying asthma experience EIB, it can also happen in people who have never had asthma before and have no symptoms other than those brought on by exercise [11]. A similar subset of patients [10] exclusively has exercise-induced asthma and not chronic daily asthma. Overall, despite extensive reporting and research on the epidemiology of asthma on a global scale, little is known about the epidemiology of exercise-induced asthma and EIB. It is now favored to define cases with underlying asthma and exercise-induced bronchoconstriction with asthma (EIBa) [12] as well as people without underlying asthma who only have symptoms when exercising as EIB. Because it can falsely indicate that exercise causes an asthma attack (rather than worsening or precipitating an attack), the term exercise-induced asthma (EIA) is no longer advised. Here, we have defined EIB as the acute onset of bronchoconstriction during or right after exercise, independent of underlying asthma in a subject. This idea represents the pathophysiology of the illness accurately. This systematic review aims mainly to investigate the recently published literature that assessed the risk of EIB among adolescent athletes with asthma.

## Review

Methodology

This systematic review was conducted in accordance with accepted standards (Preferred Reporting Items for Systematic Reviews and Meta-Analyses, PRISMA). The study duration is Between January and February 2023. To locate the pertinent literature, a detailed search was performed across six major databases, including PubMed, Web of Science, Science Direct, EBSCO, Scopus, Wiley, and Cochrane Library. We only searched in English and took into account each database's specific requirements. The studies corresponding to the following keywords were located using PubMed Medical Subject Headings (MeSH) terms; "exercise-induced asthma", " exercise-induced bronchoconstriction", "airway hyperresponsiveness", "athletes", "adolescent", "teenage", "young", and "school-age". The Boolean operators "OR" and "AND" were used to match the required keywords. The search returned previous studies with full English text, free papers, and human trials. We included only the studies that investigated the possibility of EIB in adolescent athletes diagnosed with asthma and free accessible studies in the English language; however, studies using adult individuals and those that fell beyond the basic parameters of our purpose were excluded. We applied Rayyan (QCRI) to detect duplicates in the output of the search strategy [13]. The researchers used a set of inclusion/exclusion criteria to refine the combined search results in order to evaluate the relevance of the titles and abstracts. The reviewers carefully examined each paper that met the criteria for inclusion. The authors talked about ways to resolve conflicts. The authorized study was uploaded using a data extraction form that had been prepared. The researchers gathered information on the studies' authors, years, study plans, nations, sports, prevalence of EIB, and primary outcomes. The Risk Of Bias In Non-randomised Studies - of Interventions (ROBINS-I) risk of bias assessment method for non-randomized trials of treatments was used to assess the quality of the included studies [14]. The seven topics that were assessed included confounding, participant selection for the study, classification of interventions, deviations from intended interventions, missing data, assessment of outcomes, and selection of the reported result.

Results

**Figure 1:**
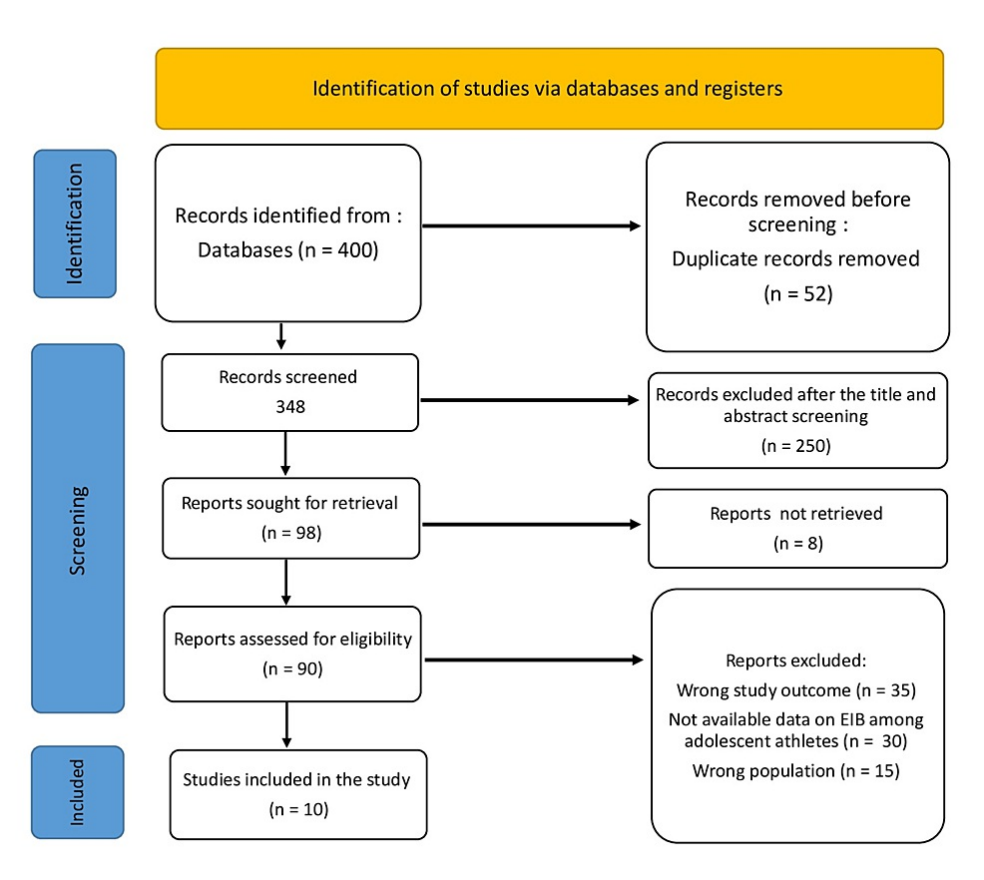
PRISMA flowchart summarizes the study selection process PRISMA: Preferred Reporting Items for Systematic Reviews and Meta-Analyses

The systematic search turned up 400 study articles in total after 52 duplicates were removed. Two hundred fifty papers were removed after 348 studies had their titles and abstracts screened. Only eight of the 98 reports that were searched for recovery could not be found. Ninety papers were eventually screened for full-text evaluation; 35 were removed due to incorrect study outcomes, 30 due to a lack of data on study outcomes, and 15 due to an incorrect population type. This systematic review contained 10 appropriate study papers. Figure 1 displays a summary of the study selection procedure. 

Characteristics of the included studies

Table 1 includes the sociodemographic characteristics. A total of ten studies with 3129 adolescent athletes were included, and their ages ranged from seven to 20 years. Three studies were conducted in the USA [15-17], one in Greece [18], one in Tunisia [19], one in Poland [20], one in Japan [21], one in Germany [22], one in Iran [23], and one in Brazil [24]. Five studies were cross-sectional studies [15, 16, 19, 21, 23], three were cohort studies [18, 22, 24], one was a prospective study [20], and one was an observational study [17].

**Table 1:** Sociodemographic characteristics of the included participants

Study	Country	Study design	Participants (n)	Age range	Males (%)
Sidiropoulou et al., 2012 [18]	Greece	Cohort	90	14 -1 8	100
Kukafka et al., 1998 [15]	USA	Cross-sectional	214	16 ± 1 (mean)	100
Aissa et al., 2009 [19]	Tunisia	Cross-sectional	196	13.5 ± 0.5 (mean)	100
Malewska-Kaczmarek et al., 2022 [20]	Poland	Prospective	101	12 - 18	61.4
Rupp et al., 1992 [16]	USA	Cross-sectional	1241	11 - 19	63
Hammerman et al., 2002 [17]	USA	Observational	801	13 -18	63
Rika et al., 2008 [21]	Japan	Cross-sectional	168	15.87 ± 1.49 (mean)	42.3
Sidiropoulou et al., 2005 [22]	Germany	Cohort	30	8 - 13	-
Ziaee et al., 2007 [23]	Iran	Cross-sectional	234	7 - 16	-
Mousinho Gomes, 2018 [24]	Brazil	Cohort	54	13 - 20	100

Table 2 presents the characteristics of the included studies. EIB diagnosis was based on exercise challenge tests, and only two were based on questionnaires [18, 22]. The prevalence of EIB among adolescent athletes ranged from 2.1% [23] to 61% [16]. Most studies have demonstrated that athletes in their adolescence suffer from EIB, which requires regular management. Two studies have reported that low-income communities [15] and humidity levels [19] are risk factors for EIB.

**Table 2:** Characteristics and outcomes of the included studies. ROBIN-I: Risk Of Bias In Non-randomised Studies - of Interventions, EIB: exercise-induced bronchoconstriction

Study	Sports	Methods for EIB diagnosis	EIB prevalence (%)	Key findings and implications	ROBIN-I
Sidiropoulou et al., 2012 [18]	Football, basketball, and water polo	Questionnaire	24.4%	Young athletes can experience EIB during a football or basketball game, but to a smaller extent than during the free running test.	Moderate
Kukafka et al., 1998 [15]	Football players	Exercise challenge test	9%	Urban varsity athletes had a high proportion of recognized EIB, which suggests that active EIB screening may be necessary to identify students at risk for EIB and asthma, especially those living in low-income neighborhoods.	Moderate
Aissa et al., 2009 [19]	Football players	Exercise challenge test	30	Teenage amateur football players in Tunisia frequently have EIB. The prevalence varies by location and appears to be influenced by air humidity levels.	Moderate
Malewska-Kaczmarek et al., 2022 [20]	Football, horse riding, tennis, dance, athletics, cycling, martial arts, gymnastics, floorball, basketball, volleyball, handball, and swimming	Exercise challenge test	28	Athletes in their adolescence frequently suffer from EIB and asthma. This group requires special attention from doctors because the symptoms can result in both under- and overdiagnosis.	Moderate
Rupp et al., 1992 [16]	Football, basketball, baseball, soccer, track/cross-country, tennis, golf, softball, cheerleading	Exercise challenge test	61	61% of students with a medical history and positive spirometry tests had EIB. The information might have an impact on more thorough screening of the adolescent population.	Moderate
Hammerman et al., 2002 [17]	Football, baseball, soccer, track, lacrosse, field hockey, and volleyball	Exercise challenge test	5.7	The high school athlete with asthma requires regular care to ensure that the prescription medicines are working. The fact that these interventions work enables the athlete to perform to their highest level.	Moderate
Rika et al., 2008 [21]	Archery, athletics, gymnastics, lawn tennis, swimming, table tennis, taekwondo, volleyball, weightlifting, badminton, basketball, soccer	Exercise challenge test	13.7	Adolescent athletes had a moderately high prevalence of EIB, which was more common in females. Additionally, a laboratory exercise challenge may cause EIB in a less asthmatic sport.	High
Sidiropoulou et al., 2005 [22]	Soccer	Questionnaire	40	When diagnosing bronchial hyperresponsiveness in young athletes, the identification of EIB by an exercise challenge test is helpful. The same research should be done on younger and older athletes who play various sports and activities.	Moderate
Ziaee et al., 2007 [23]	Soccer	Exercise challenge test	2.1	EIB is present in at least 2.1% of soccer players with no history of allergies or asthma. This value indicates that even if a soccer player has never had asthma or allergies, there is still a high likelihood that they may experience bronchospasm.	High
Mousinho Gomes, 2018 [24]	Soccer	Exercise challenge test	7	Low BIE prevalence was found in the group of semi-pro soccer players who had no exercise-related respiratory symptoms. It can be required for athletes who also compete in more arid and chilly areas to complete the challenge while breathing dry air.	Moderate

Discussion

Asthma can become particularly troublesome during adolescence. Managing a chronic illness during adolescence might interfere with normal growth and the physical, cognitive, emotional, and psychological changes brought on by puberty. It is important to carefully consider how these changes affect the pathophysiology, presentation, prognosis, and therapeutic options for asthma [25].

This systematic review investigated the published literature on EIB among adolescent athletes with asthma. Most studies have demonstrated that athletes in their adolescence suffer from EIB, which requires regular management. We reported a prevalence of EIB that ranged from 2.1% [23] to 61% [16]. This variance in the prevalence of EIB is unexplainable due to the qualitative nature of the study. However, this heterogeneity could be attributable to the variable sample sizes, ethnic factors, environmental factors, and nature of study designs.

Similarly, Del Giacco et al. [26] reported that although asthma-related symptoms are frequent in high school and adolescent athletes, their occurrence raises a number of complicated issues regarding the diagnosis of asthma, its clinical management, adherence to treatment, and its effects on the quality of life (QOL). Some athletes might not have received the proper screening and could be going through silent episodes of EIB, which could lead to long-term respiratory health issues. Many athletes might neglect to mention symptoms that could be EIB and refuse to go to the doctor for a formal diagnosis. This might be explained by athletes' ignorance of symptoms and their connection to EIB. Athletes may mistake their symptoms for a bad day of training or a lack of conditioning rather than realizing they could have EIB.

Another review reported that athletes frequently complain of coughing or wheezing during or right after exercise when they visit a doctor. During practice or competition, they might express complaints about having trouble keeping up with their teammates or feeling out of breath. Despite the fact that more severe cardiopulmonary conditions should always be kept in mind when making a diagnosis, EIB is very common in both the general population and athletes [27].

Our study reported some risk factors, including low-income communities [15] and humidity levels [19] are for EIB. Greater asthma severity (mortality and hospitalization rates) and prevalence have previously been linked to indicators of poverty [28], such as lower income, living in densely populated areas with poor healthcare access and utilization patterns, and living in inner cities. Urban poverty areas have a variety of environmental factors that can affect care-seeking behavior [28, 29]. Koh and Choi [30] also reported that relative humidity is a significant factor in the development of EIB. Humidity can probably be used to explain the differences in EIB between subjects in various cities across the nation.

The majority of young adults with asthma have positive self-images [31]. Adolescents with asthma are more likely to resent their illness and feel different from their peers, which makes them ignore or hate having asthma. Adolescents with asthma participate in the same risky behaviors as their peers, like smoking [32]. Teenagers are especially vulnerable during this time because they are increasingly in charge of their asthma management, despite the fact that they frequently overestimate and underestimate the severity of their condition, respectively [33].

Additionally, asthma can affect a person's social and academic life, which frequently leads to isolation. In order to avoid social isolation and being labeled as different, some asthmatics purposefully hide their symptoms and "struggle through" physical activity with their healthy peers [34]. Teenagers occasionally view their symptoms as a "normal" reaction to exercise or prefer to conceal them, perhaps out of a fear of being kicked off their team. Recent research found only a slight difference in how bronchoconstriction-related symptoms were perceived by athletes and non-athletes (aged 14-35), with the younger group experiencing worse perception [35, 36]. As a result, relying solely on symptoms to identify asthma in young athletes will almost certainly result in underdiagnosis.

Finally, the included studies had relatively small sample sizes, resulting in low power to our results. We were unable to conduct analyses to explore potential bias associated with the heterogeneity between studies because of the small number of included studies. The majority of the included studies used cross-sectional, which reduces our confidence in these results because bias can be introduced.

## Conclusions

This systematic review demonstrated that EIB is frequent among adolescent athletes. The prevalence varies between countries due to different social and environmental factors. There is a need for better management and improved detection of respiratory conditions in young athletes, as well as better respiratory care provision and avoidance of triggering factors and certain medications. Therefore, it is necessary to move away from a symptom-based diagnosis of EIB/EIBa and incorporate objective testing through indirect bronchial provocation through exercise or its substitutes. Changes should be driven by policies in order to guarantee the success of new initiatives. Education programs created for young athletes and their support staff should be used to support guidelines (including coaches, team doctors, physiotherapists, etc.). Finally, in order to establish the risk factors for EIB and maybe develop biomarkers to identify young athletes at risk, longitudinal follow-up of young athletes is required. The development of a step-by-step process for testing young and teenage athletes for exercise-induced bronchoconstriction. Consider age, sports discipline, and the number of hours played each week when determining which of the young athletes would benefit from a screening test to identify young athletes at risk.
